# Molten Globule-Like Partially Folded State of *Bacillus licheniformis *α**-Amylase at Low pH Induced by 1,1,1,3,3,3-Hexafluoroisopropanol

**DOI:** 10.1155/2014/824768

**Published:** 2014-04-07

**Authors:** Adyani Azizah Abd Halim, Mohammed Suleiman Zaroog, Habsah Abdul Kadir, Saad Tayyab

**Affiliations:** ^1^Biomolecular Research Group, Biochemistry Programme, Institute of Biological Sciences, Faculty of Science, University of Malaya, 50603 Kuala Lumpur, Malaysia; ^2^Faculty of Applied Medical Sciences, University of Gezira, P.O. Box 20, 2667 Wad Medani, Sudan

## Abstract

Effect of 1,1,1,3,3,3-hexafluoroisopropanol (HFIP) on acid-denatured *Bacillus licheniformis *α**-amylase (BLA) at pH 2.0 was investigated by far-UV CD, intrinsic fluorescence, and ANS fluorescence measurements. Addition of increasing HFIP concentrations led to an increase in the mean residue ellipticity at 222 nm (MRE_222 nm_) up to 1.5 M HFIP concentration beyond which it sloped off. A small increase in the intrinsic fluorescence and a marked increase in the ANS fluorescence were also observed up to 0.4 M HFIP concentration, both of which decreased thereafter. Far- and near-UV CD spectra of the HFIP-induced state observed at 0.4 M HFIP showed significant retention of the secondary structures closer to native BLA but a disordered tertiary structure. Increase in the ANS fluorescence intensity was also observed with the HFIP-induced state, suggesting exposure of the hydrophobic clusters to the solvent. Furthermore, thermal denaturation of HFIP-induced state showed a non-cooperative transition. Taken together, all these results suggested that HFIP-induced state of BLA represented a molten globule-like state at pH 2.0.

## 1. Introduction

Various noncovalent forces such as hydrogen bonding, electrostatic interactions, and hydrophobic interactions are known to stabilize the native protein structure [1, 2]. The information about how and when different noncovalent forces assemble to guide protein folding can be obtained from the studies of the folding intermediates and denatured states obtained under non-native conditions such as high ionic strength, extremes of pH, organic solvents, varied temperatures, or mild denaturing conditions [3, 4]. At extreme pH, various proteins are denatured due to the destabilizing repulsive forces between similar charges [5–7], whereas the attainment of the non-native states of proteins can be easily made with solvent perturbation [8–10].

Alcohols are widely used to induce secondary structure formation by stabilizing the *α*-helical structure and reducing the exposure of peptide backbone in the unfolded proteins [11, 12]. A halogenol, 1,1,1,3,3,3-hexafluoroisopropanol (HFIP), a fluorine substituted alcohol is known to exhibit highest *α*-helix inducing potential in many proteins [13–15]. Furthermore, low concentrations of both HFIP and 2,2,2-trifluoroethanol (TFE) have been found to produce a molten globule-like state in acid-denatured proteins [16, 17]. The molten globule state is considered to provide the information about the early stages in the folding pathway where there are no specific interactions between the side chain residues of the protein [18, 19].

*Bacillus licheniformisα*-amylase (BLA), a highly thermostable enzyme [20], has been widely used in various industries involving high temperature conditions such as in the production of maltodextrin and alcohols, baking, textile, and detergent industries for the initial starch hydrolysis [21–25]. Since BLA is known to possess higher thermal stability compared to* Bacillus amyloliquefaciensα*-amylase (BAA) which has 81% identity and 88% similarity towards BLA, it is valuable to understand the folding mechanism and structural-stability of this enzyme [26]. Acid denaturation of BLA has been shown to complete at pH 2.0 [27]. However, the molten globule state of BLA has been characterized at pH 4.0 by various probes and it has been found more stable than pH 4.0 state of BAA upon guanidine hydrochloride denaturation and proteolytic digestion [27]. Moreover, a partially folded state has been found to accumulate at higher TFE concentrations in both native BLA and BAA but without the characteristics of a molten globule state [28]. Although many attempts have been made to elucidate the structure-stability relationship of BLA, effect of HFIP on the acid-denatured state of BLA at pH 2.0 has not been attempted so far. In this report, we present our data on HFIP-induced conformational transition in the acid-denatured BLA at pH 2.0 using different probes such as far-UV and near-UV CD, intrinsic fluorescence, ANS fluorescence, and acrylamide quenching.

## 2. Materials and Methods

### 2.1. Materials

*Bacillus licheniformisα*-amylase (BLA) (Catalog no. A4551), 8-anilino-1-naphthalene sulfonic acid (ANS) (Catalog no. A3125), acrylamide (Catalog no. A8887),* N*-acetyl-L-tryptophanamide (NATA) (Catalog no. A6501), and 1,1,1,3,3,3-hexafluoro-2-propanol (HFIP) (Catalog no. 105228) were purchased from Sigma-Aldrich Inc., St. Louis, MO, USA. Tris base was supplied by Amresco, Irvine, CA, USA. All other chemicals used were of analytical grade purity.

### 2.2. Analytical Procedures

BLA concentration was determined spectrophotometrically on a Shimadzu double-beam spectrophotometer, model UV-2450, using a molar extinction coefficient of 139,690 M^−1^ cm^−1^ at 280 nm [29] whereas a molar extinction coefficient of 5000 M^−1^ cm^−1^ at 350 nm was used to measure ANS concentration [30]. NATA concentration was determined using a molar extinction coefficient of 5630 M^−1^ cm^−1^ at 280 nm [31].

### 2.3. Circular Dichroism

Circular dichroism (CD) measurements were performed at 25°C on a Jasco spectropolarimeter, model J-815, equipped with a thermostatically-controlled cell holder under constant nitrogen flow after calibrating the instrument with (+)-10-camphorsulfonic acid. The CD spectra were recorded in the far-UV (200–250 nm) and the near-UV (250–300 nm) regions using a protein concentration and cuvette path length of 1.7 *μ*M; 1 mm, and 3.4 *μ*M; 10 mm, respectively. A scan speed of 100 nm/min and a response time of 1 s were employed throughout the spectral measurements. Each spectrum was the average of three scans and the spectra were corrected with suitable blanks after subtracting the CD spectral contribution of the blank solutions from the CD spectra of the protein samples. The results are expressed as mean residue ellipticity (MRE) in deg·cm^2^·dmol^−1^, which was obtained using the following equation:

(1) $$\begin{array}{c} \text{MRE}=\frac{\theta \times \text{MRW}}{10\;\times c\times l}, \end{array}$$

where different terms, such as *θ*, MRW, *c*, and *l* refer to ellipticity in millidegrees, mean residue weight (molecular weight, 55200 Da/number of amino acid residues, 483) of the protein, protein concentration in mg/mL, and the optical path length in cm, respectively. Calculation of helical content was made using the method of Chen et al. [32].

### 2.4. Fluorescence Spectroscopy

Intrinsic fluorescence measurements were made in the wavelength range, 300–400 nm upon excitation at 280 nm, on a Hitachi fluorescence spectrophotometer, model F-2500 using a protein concentration of 0.1 *μ*M in a 1 cm path length cuvette. Both excitation and emission slits were fixed at 10 nm each.

ANS fluorescence spectra of the sample containing ANS (30 *μ*M) and protein (0.6 *μ*M) in a ratio of 50 : 1 were recorded in the wavelength range, 400−600 nm upon excitation at 380 nm. The fluorescence spectra were corrected for the fluorescence contribution of the blank solutions.

### 2.5. Preparation of Native and Acid-Denatured BLAs

Native BLA solution was prepared by dissolving the protein in 10 mM Tris-HCl buffer, pH 7.0, whereas 10 mM glycine-HCl buffer, pH 2.0, was used to prepare acid-denatured BLA. This solution was incubated at 4°C for 12 hours and filtered through 0.45 *μ*m millipore filter before use.

### 2.6. HFIP-Induced Structural Transitions

Titration of acid-denatured BLA with increasing HFIP concentrations was carried out by adding increasing concentrations (0–6.0 M) of HFIP to a constant volume (0.5 mL) of the protein solution taken in different tubes. The total volume of the mixture was made to 5.0 mL with 10 mM glycine-HCl buffer, pH 2.0. Blank solutions were prepared in the same way except that the protein solution was substituted with the same volume of the above buffer. The pH of the incubation mixture was found to remain within ±0.1 pH unit. The CD and fluorescence spectral measurements were made after 30 min incubation at 25°C, as suggested earlier [14].

For ANS-binding experiments, 0.5 mL of ANS stock solution (300 *μ*M) was added to the incubation mixture containing fixed concentration of the protein (0.6 *μ*M) and varying HFIP concentrations (0–6.0 M), which were preincubated at 25°C for 30 min. The sample incubation mixture (5.0 mL) was incubated for additional 20 min before spectral measurements. Blank solutions without protein were prepared in the same way except for substituting protein with the buffer and their fluorescence contribution (if any) was subtracted from the fluorescence spectra of the protein.

### 2.7. Acrylamide Quenching

Acrylamide quenching experiments were performed on a Hitachi fluorescence spectrophotometer, model F-2500, using a concentration of 0.1 *μ*M and 1.7 *μ*M for BLA and NATA, respectively. Increasing volumes of acrylamide stock solution (2.0 M) were added to 0.5 mL protein solution (0.6 *μ*M) of different BLA samples (native and acid-denatured BLAs both in the absence and the presence of 0.4 M HFIP) or NATA solution to get the desired acrylamide concentration in the range, 0.02–0.25 M. The total volume (3.0 mL) was made with the respective buffers and the final incubation mixture was kept in dark for 30 min before fluorescence measurements. Emission spectra were recorded in the wavelength range, 300–400 nm, upon excitation at 295 nm in order to excite the tryptophan (Trp) residues only, using slit widths of 10 nm. The fluorescence intensity values of each sample were corrected with respective blanks and the data were analyzed following the Stern-Volmer equation as described earlier [33].

### 2.8. Thermal Denaturation

Effect of temperature (25–100°C) on different states of BLA (native, acid-denatured, and HFIP-induced states) was studied using CD spectral change at 222 nm with a scan rate of 1.0°C/min. Transformation of ellipticity values into MRE values was made in the same way as described above.

## 3. Results and Discussion

### 3.1. HFIP-Induced Structural Transitions in the Acid-Denatured BLA

**Figure 1:**
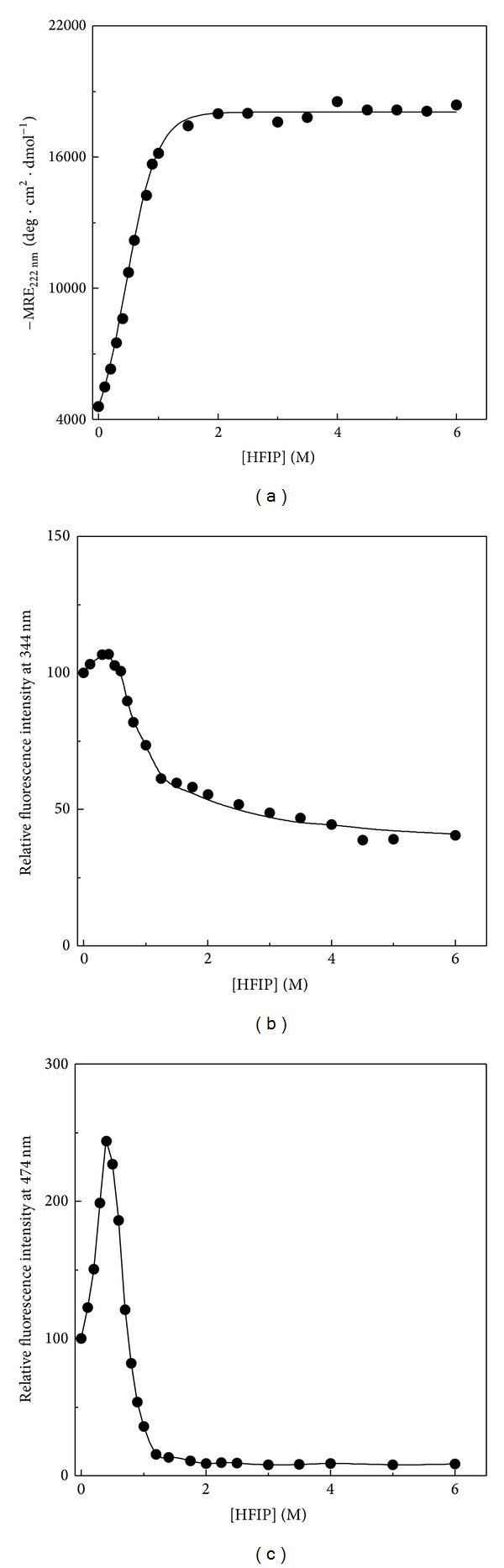
Effect of increasing HFIP concentrations on the spectral characteristics of the acid-denatured BLA at pH 2.0 and 25°C, as monitored by (a) MRE_222 nm_ measurements, (b) intrinsic fluorescence measurements at 344 nm upon excitation at 280 nm, and (c) ANS fluorescence measurements at 474 nm upon excitation at 380 nm.

Figure 1 shows the effect of increasing HFIP concentrations on the acid-denatured BLA at pH 2.0 as monitored by MRE_222 nm_ (a), intrinsic fluorescence (b), and ANS fluorescence (c) measurements. As can be seen from Figure 1(a), a marked increase in the −MRE_222 nm_ value of the acid-denatured BLA was observed on increasing the HFIP concentration up to 1.5 M, beyond which it sloped off. Similarly, increase in both intrinsic fluorescence and ANS fluorescence intensities was observed up to 0.4 M HFIP, being more significant with ANS fluorescence (2.4-fold) compared to intrinsic fluorescence. Further increase in the HFIP concentration led to a gradual decrease in both fluorescence intensities, tailing off around 1.2 M HFIP. Increase in the intrinsic fluorescence intensity at early HFIP concentration (up to 0.4 M HFIP) may be attributed to the change in the microenvironment around tyrosine (Tyr) and tryptophan (Trp) residues due to the separation of negatively charged residues (quenchers) such as glutamate or aspartate from the vicinity of the aromatic residues [34]. This seems possible in view of the increase in the secondary structures of the acid-denatured BLA as shown in Figure 1(a). On the other hand, presence of trifluoromethyl groups in the fluorinated alcohols may account for the observed decrease in the fluorescence intensity at higher HFIP concentrations. Both high electronegativity of fluorine (F) atoms and large field effects of trifluoromethyl groups can make these fluoroalcohols as active proton donors [35, 36], which may result in the quenching of Trp fluorescence due to excited state proton transfer from fluorinated alcohols to indole ring of Trp residues [35].

Marked increase in the ANS fluorescence intensity, as observed up to 0.4 M HFIP, reflected the formation of a compact denatured state with a large number of surface-exposed hydrophobic clusters [5], which were otherwise absent in the acid-denatured state. This was in accordance with our intrinsic fluorescence results, where HFIP-induced state showed some burial of aromatic chromophores. Decrease in the ANS fluorescence intensity beyond 0.4 M HFIP reflected the disruption of the hydrophobic clusters. In view of the increased MRE_222 nm_ value and increased ANS binding observed at 0.4 M HFIP, it appears that the acid-denatured BLA might be able to form a molten globule-like state in the presence of 0.4 M HFIP.

### 3.2. Characterization of the HFIP-Induced State

The HFIP-induced state obtained at 0.4 M HFIP was characterized using far- and near-UV CD, intrinsic fluorescence and ANS fluorescence spectra, acrylamide quenching, and thermal denaturation studies.

#### 3.2.1. Far-UV CD Spectra

**Figure 2:**
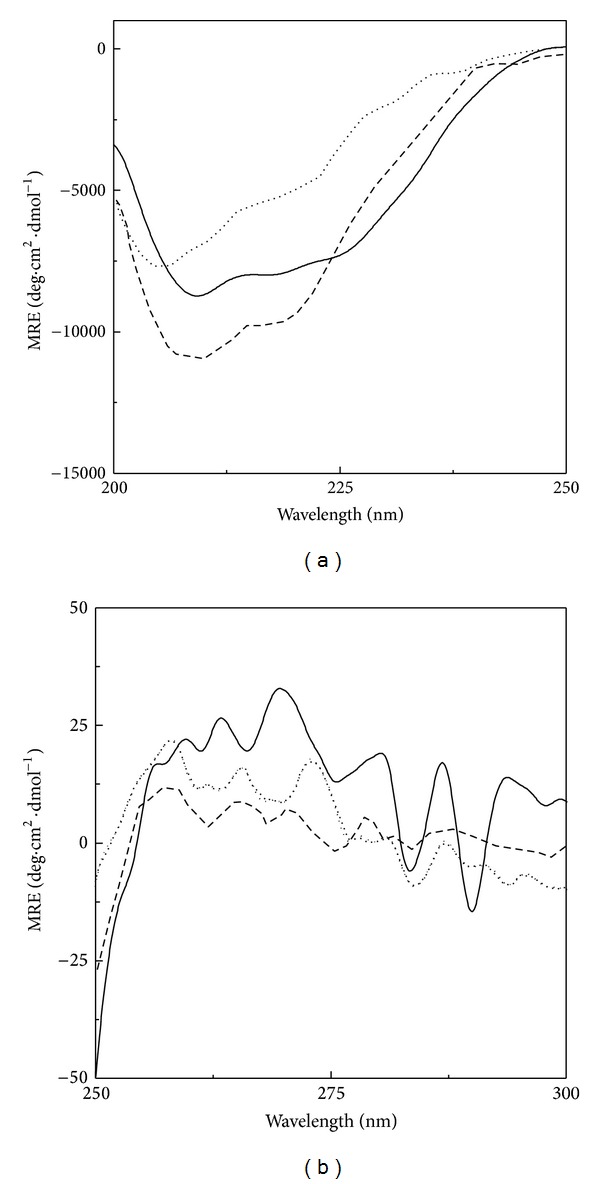
Far-UV (a) and near-UV (b) CD spectra of different conformational states of BLA, as obtained at 25°C using a protein concentration and cuvette path length of 1.7 *μ*M; 1 mm and 3.4 *μ*M; 10 mm for far-UV and near-UV CD spectral measurements, respectively. Native state (—), acid-denatured state (···), and HFIP-induced state (- - -).

A comparison of secondary structural characteristics of different states of BLA was made using far-UV CD spectroscopy.  Figure 2(a) shows far-UV CD spectra of the native, the acid-denatured, and the HFIP-induced states of BLA. As evident from the figure, native state of BLA was characterized by the presence of two minima around 208 and 222 nm, characteristic of *α*-helical structure [28, 37], which were shifted towards lower wavelength side at 205 and 220 nm along with reduced MRE values in the acid-denatured state (Figure 2(a)). A significant reduction (~38%) in the MRE_222 nm_ value in the acid-denatured state indicated significant loss of the secondary structures (Table 1). Interestingly, far-UV CD spectrum of the HFIP-induced state was found closer to the far-UV CD spectrum of the native protein, showing ~85% increase in the MRE_222 nm_ value from that obtained with the acid-denatured state, being −8615 compared to −7557, obtained with the native BLA (Table 1). A quantitative analysis of these spectra was made by calculating the percentage *α*-helical content for these states. The value of the *α*-helical content decreased from ~17% (for native BLA) to ~8% in the acid-denatured state, showing significant loss of the secondary structure but regained further in the HFIP-induced state, showing ~21% *α*-helical content (Table 1). This result was in accordance with earlier observations, as alcohols are known to increase *α*-helical structure by promoting local polar interactions such as hydrogen bonds in proteins [12, 38]. Such effect has been found more with fluoroalcohols compared to alkyl alcohols [14, 15].

#### 3.2.2. Near-UV CD Spectra

Near-UV CD spectrum provides information about the protein's tertiary structure.  Figure 2(b) shows near-UV CD spectra of different states of BLA. Several positive signals (maxima) and two minima around 283 and 290 nm characterized the near-UV CD spectrum of native BLA, suggesting fixed orientation of various aromatic residues under native conditions. These near-UV CD spectral characteristics of BLA were similar to those reported earlier [28]. Significant alteration in the near-UV CD spectrum of the acid-denatured BLA, particularly in the wavelength range, 260–300 nm suggested more flexible orientation of the majority of the aromatic residues. As can be seen from the figure, some regions of BLA were significantly affected, while others showed least alteration in the acid-denatured state, suggesting partial denaturation. This was in accordance with previous reports on the acid-denatured proteins, showing the presence of significant residual structure [39, 40]. Presence of 0.4 M HFIP did not produce any significant alteration in the tertiary structure of the acid-denatured BLA. These results indicated that the HFIP-induced state retained similar secondary structures as that found in native BLA but a disordered tertiary structure, suggesting the characteristics of the molten globule-like state [41, 42].

#### 3.2.3. Intrinsic Fluorescence Spectra

**Figure 3:**
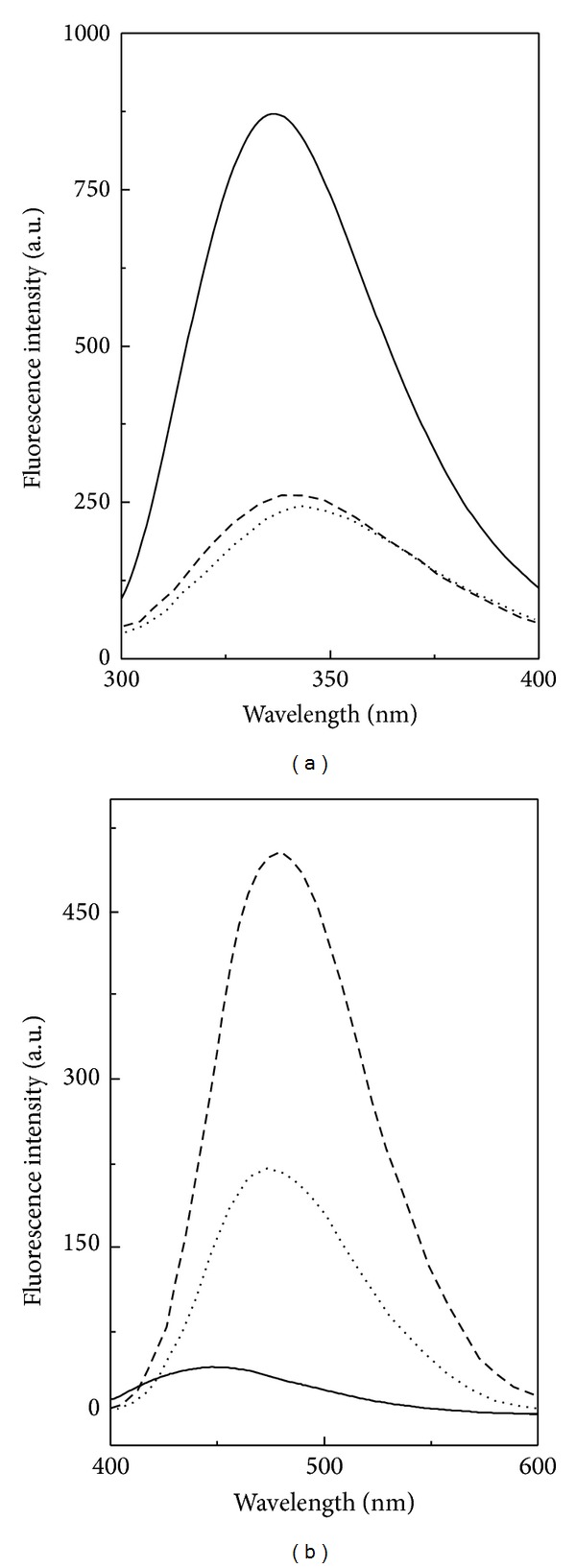
Intrinsic fluorescence (a) and ANS fluorescence (b) spectra of different conformational states of BLA, as obtained at 25°C using a protein concentration of 0.1 *μ*M and 0.6 *μ*M for intrinsic fluorescence and ANS fluorescence measurements, respectively. Native state (—), acid-denatured state (···), and HFIP-induced state (- - -).

The information about the tertiary structural alteration in a protein can also be obtained from its intrinsic fluorescence spectra. The intrinsic fluorescence spectrum of native BLA (Figure 3(a)) was characterized by the presence of an emission maximum around 337 nm due to the abundance of Trp residues [43]. BLA has a total of 17 Trp and 31 Tyr residues [44]. A marked decrease (~72%) in the fluorescence intensity accompanied by a significant red shift (7 nm) from 337 nm to 344 nm was observed in the intrinsic fluorescence spectrum of the acid-denatured BLA (Figure 3(a), Table 1). These results indicated the change in the microenvironment around Tyr and Trp residues from nonpolar to polar, as a result of partial unfolding of the protein in the acid-denatured state [45]. A small increase (~7%) in the fluorescence intensity accompanied by a blue shift of 3 nm characterized the fluorescence spectrum of the HFIP-induced state. Such small changes in the fluorescence characteristics of the HFIP-induced state indicated slight internalization of Trp and Tyr residues to a more hydrophobic environment due to local structural perturbation [17, 34]. These results were similar to the near-UV CD spectral results, showing little alteration in the tertiary structure of the HFIP-induced state. In other words, the tertiary structure of HFIP-induced state remained disorganized.

#### 3.2.4. ANS Fluorescence Spectra

ANS binding is used to probe the exposure of the hydrophobic clusters in proteins [46]. Upon binding to the hydrophobic regions in the protein, ANS produces a fluorescence spectrum in the wavelength range, 400–600 nm upon excitation at 380 nm. ANS has shown greater affinity towards the acid-denatured state than the native state of proteins, suggesting exposure of the buried hydrophobic patches to the solvent in the acid-denatured state [27, 47, 48]. The ANS fluorescence spectra of different states of BLA are shown in Figure 3(b). ANS binding to native BLA produced a weak fluorescence signal with an emission maximum at 448 nm, suggesting burial of the hydrophobic ANS binding clusters in the protein interior. A marked increase (~6-fold) in the ANS fluorescence intensity along with a significant red shift (26 nm) in the emission maximum, observed with the acid-denatured BLA, indicated exposure of the solvent accessible hydrophobic regions and agreed well with the previous reports on other proteins [49, 50]. Interestingly, ANS fluorescence spectrum of the HFIP-induced state showed ~2.4-fold increase in the ANS fluorescence intensity accompanied by a 3 nm red shift from that obtained with the acid-denatured state, suggesting more exposure of the ANS binding sites (more hydrophobic clusters) to the solvent (Figure 3(b), Table 1) [48].

#### 3.2.5. Acrylamide Quenching

**Figure 4:**
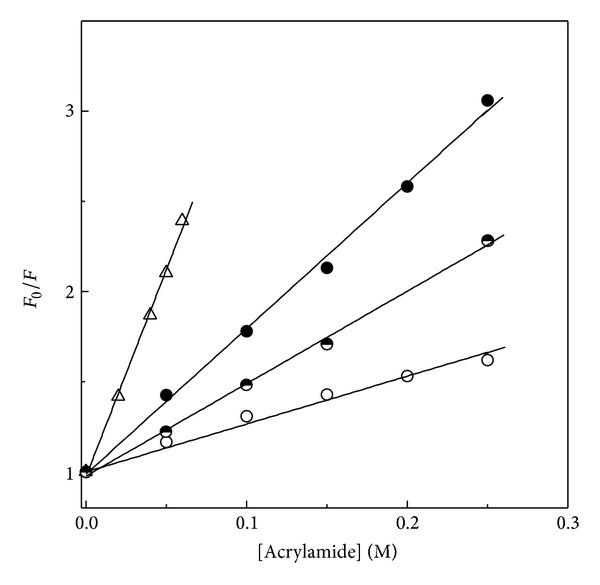
Stern-Volmer plots of different conformational states of BLA, as studied by acrylamide quenching using a protein concentration of 0.1 *μ*M. Native state (◯), acid-denatured state (●), and HFIP-induced state (*◓*). Acrylamide quenching data obtained with NATA is shown by symbols (△).

Acrylamide quenching studies were also made to evaluate the exposure of Trp residues in different states of BLA. The results were fitted into the Stern-Volmer plots (Figure 4) and values of the Stern-Volmer constant, *K*_SV_, obtained from the slope of the linear plots, are given in Table 2. For the complete exposure of Trp residues, data of NATA was used as a reference. The *K*_SV_ value of NATA (22.8 M^−1^) was found to be significantly higher than the value obtained for different states of BLA. This value was similar to the *K*_SV_ value reported for NATA in several earlier reports [51, 52]. *K*_SV_ value (8.1 M^−1^) of the acid-denatured BLA was found higher than the native BLA (2.5 M^−1^) but much lower than the *K*_SV_ value obtained for NATA, suggesting more exposure of Trp residues in the acid-denatured state compared to native BLA. However, a significantly lower value of *K*_SV_ compared to NATA suggested that acid denaturation did not unfold the protein completely [53]. A marked quenching of the intrinsic fluorescence compared to the native protein has also been reported for BAA at pH 2.25 [54]. Decrease in the *K*_SV_ value from 8.1 M^−1^ (for the acid-denatured BLA) to 5.1 M^−1^ (for the HFIP-induced state) clearly suggested burial of Trp residues due to local structural changes [43]. In other words, HFIP-induced state showed internalization of Trp residues to the non-polar environment compared to the acid-denatured BLA and agreed well with a previous report on stem bromelain, where presence of HFIP has been shown to decrease the *K*_SV_ value compared to the acid-denatured protein [17].

#### 3.2.6. Thermal Denaturation

**Figure 5:**
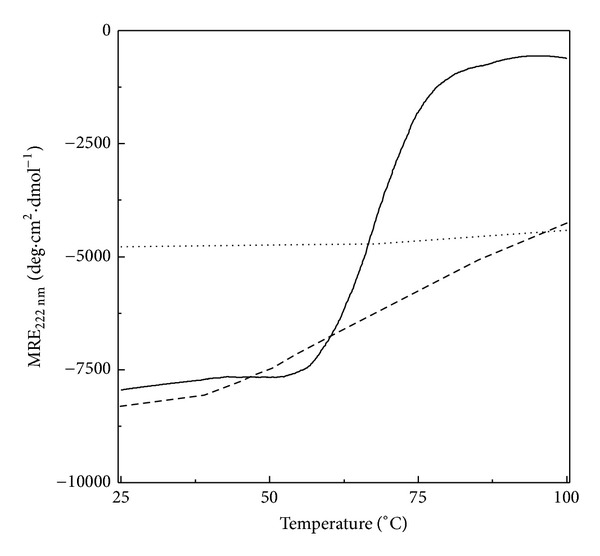
Temperature dependence of the far-UV CD spectral signal, MRE_222 nm_ of different conformational states of BLA. Native state (—), acid-denatured state (···), and HFIP-induced state (- - -).

Figure 5 shows thermal denaturation curves of different states of BLA, as obtained from MRE_222 nm_ measurements. Thermal denaturation of native BLA showed a cooperative transition, characterized by a sharp decrease in −MRE_222 nm_ within the temperature range, 60–77°C with the occurrence of the melting temperature (*T*_*m*_) value at 68°C. This agreed well with the reported T_m_ value of BLA, obtained from DSC experiments [55]. The cooperative thermal transition of the native BLA has also been shown in an earlier study [56]. On the other hand, the acid-denatured BLA did not produce any significant change in the MRE_222 nm_ value throughout the temperature range, indicating thermal stability of the enzyme in the acid-denatured state with respect to the *α*-helical content. It appears that the acid-denatured state might be similar to the thermal-denatured state in terms of stability. In a previous report, Zaroog and Tayyab [57] have also shown thermal stability of the acid-denatured glucoamylase within the temperature range, 25–100°C. Interestingly, acid-denatured BLA in the presence of 0.4 M HFIP (HFIP-induced state), though retained similar secondary structure as that found in native BLA state (Figure 2(a)) but showed a non-cooperative thermal transition (Figure 5, Table 1), a characteristic feature of the molten globule state [58, 59]. Formation of a compact-denatured conformation of cytochrome c at pH 1.9 has also been shown to be accumulated in the presence of HFIP [16].

## 4. Conclusion

In summary, HFIP-induced state of BLA was found to possess the characteristics of a molten globule state, showing a native-like secondary structure as evident from the far-UV CD spectra, a disrupted tertiary structure as reflected from the near-UV CD spectra and intrinsic fluorescence spectra, increased ANS binding, and non-cooperative thermal transition.

## Figures and Tables

**Table 1:** Physical characteristics of different conformational states of BLA.

Variable	Native state	Acid-denatured state	HFIP-induced state
MRE_222 nm_ (deg·cm^2^·dmol^−1^)	−7557	−4649	−8615
% Helix^a^	~17	~8	~21
Fluorescence intensity	871.2	241.8	259.8
Emission maximum (*λ*_ex_ = 280 nm)	337 nm	344 nm	341 nm
ANS fluorescence intensity	33	200	481
Emission maximum (*λ*_ex_ = 380 nm)	448 nm	474 nm	477 nm
Cooperativity (thermal transition)	Yes	No	No

^a^Calculated by the method of Chen et al. [32].

**Table 2:** Values of Stern-Volmer constant (*K*_SV_) of different conformational states of BLA, as obtained from acrylamide quenching data.

BLA	*K*_SV_ (M^−1^)
Native state	2.5
Acid-denatured state	8.1
HFIP-induced state	5.1
NATA	22.8
